# Misinformation, perceptions towards COVID-19 and willingness to be vaccinated: A population-based survey in Yemen

**DOI:** 10.1371/journal.pone.0248325

**Published:** 2021-10-29

**Authors:** Ahmad Naoras Bitar, Mohammed Zawiah, Fahmi Y. Al-Ashwal, Mohammed Kubas, Ramzi Mukred Saeed, Rami Abduljabbar, Ammar Ali Saleh Jaber, Syed Azhar Syed Sulaiman, Amer Hayat Khan

**Affiliations:** 1 Discipline of Clinical Pharmacy, School of Pharmaceutical Sciences, Universiti Sains Malaysia, Penang, Malaysia; 2 Department of Pharmacy Practice, College of Clinical Pharmacy, University of Al Hodeida, Al Hodeida, Yemen; 3 Clinical Pharmacy Department, University of Science and Technology Hospital, Sana’a, Yemen; 4 Pharmacy Practice Department, Kulliyyah of Pharmacy, International Islamic University Malaysia, Kuantan, Pahang, Malaysia; 5 Department of Clinical Pharmacy and Pharmacy Practice, University of Science and Technology, Sana’a, Yemen; 6 Department of Pharmaceutical sciences, School of Pharmacy, The University of Jordan, Amman, Jordan; 7 Department of Biopharmaceutics and Clinical Pharmacy, School of Pharmacy, The University of Jordan, Amman, Jordan; 8 Department of Clinical Pharmacy & Pharmacotherapeutics, Dubai Pharmacy College for Girls, Dubai, United Arab Emirates; Uniwersytet Zielonogorski, POLAND

## Abstract

**Background:**

Since the beginning of the COVID-19 outbreak, many pharmaceutical companies have been racing to develop a safe and effective COVID-19 vaccine. Simultaneously, rumors and misinformation about COVID-19 are still widely spreading. Therefore, this study aimed to investigate the prevalence of COVID-19 misinformation among the Yemeni population and its association with vaccine acceptance and perceptions.

**Methods:**

A cross-sectional online survey was conducted in four major cities in Yemen. The constructed questionnaire consisted of four main sections (sociodemographic data, misinformation, perceptions (perceived susceptibility, severity, and worry), and vaccination acceptance evaluation). Subject recruitment and data collection were conducted online utilizing social websites and using the snowball sampling technique. Descriptive and inferential analyses were performed using SPSS version 27.

**Results:**

The total number of respondents was 484. Over 60% of them were males and had a university education. More than half had less than 100$ monthly income and were khat chewers, while only 18% were smokers. Misinformation prevalence ranged from 8.9% to 38.9%, depending on the statement being asked. Men, university education, higher income, employment, and living in urban areas were associated with a lower misinformation level (*p* <0.05). Statistically significant association (*p* <0.05) between university education, living in urban areas, and being employed with perceived susceptibility were observed. The acceptance rate was 61.2% for free vaccines, but it decreased to 43% if they had to purchase it. Females, respondents with lower monthly income, and those who believed that pharmaceutical companies made the virus for financial gains were more likely to reject the vaccination (*p* <0.05).

**Conclusion:**

The study revealed that the acceptance rate to take a vaccine was suboptimal and significantly affected by gender, misinformation, cost, and income. Furthermore, being female, non-university educated, low-income, and living in rural areas were associated with higher susceptibility to misinformation about COVID-19. These findings show a clear link between misinformation susceptibility and willingness to vaccinate. Focused awareness campaigns to decrease misinformation and emphasize the vaccination’s safety and efficacy might be fundamental before initiating any mass vaccination in Yemen.

## Introduction

More than one year has passed since the beginning of the severe acute respiratory syndrome coronavirus-2 (SARS-CoV-2) pandemic. At the same time, more than 30 biotech and pharmaceutical companies were racing to develop a safe and effective COVID-19 vaccine. The World Health Organization (WHO) has approved eight vaccines for emergency use; the Pfizer-BioNTech, AstraZeneca, Moderna, Sinopharm, Sinovac, and Johnson & Johnson vaccines [1, 2]. Large-scale vaccination programs are planned to reach herd immunity against COVID-19. However, such a program’s success will majorly depend on the public response toward the vaccine.

Vaccination in Yemen should be of utmost priority because this country lacks in terms of medical infrastructure [3]. As of March 2021, Yemen has received around 360000 doses of AstraZeneca vaccine from the COVID-19 Vaccines Global Access Facility (COVAX), and it is expecting to get 14 million doses of COVID-19 vaccines under the same initiative, covering 23% of the population [4]. However, vaccination in Yemen is not obligatory, and as of August 26, 2021, only 311,483 vaccination doses have been delivered [5]. Vaccination acceptance can reflect the general perception about COVID-19 and its newly developed vaccines, and the increased awareness of the risk of COVID-19 and the benefits of vaccination can increase the general uptake of the vaccine [6].

There are vital factors that might influence the public response and acceptance of the newly developed vaccines, like perceived susceptibility and severity towards COVID-19 and the misinformation spread [7, 8]. Doubts about the vaccine effectiveness, safety, and usefulness are also major obstacles for the population acceptance of COVID-19 vaccines worldwide. For example, in France, 25% of the population reported refusing the vaccine due to safety concerns [9]. In Saudi Arabia, 36% showed no interest in the vaccine [10], while in the USA, the Food and Drug Administration’s (FDA) emergency use authorization was associated with a lower probability of accepting the vaccine [11]. In the UK, the willingness to be vaccinated was linked to more positive general vaccination beliefs and attitudes and weaker beliefs about the vaccine being unsafe or it causes severe side effects; also, well-informed subjects had a positive perception about the benefits of the vaccine and the danger of COVID-19 [6].

To the best of our knowledge, no previous research in the Middle East assessed the association between misinformation and willingness to vaccinate. In addition, the conducted research about COVID-19 in Yemen is exceptionally scarce. In a recent study, we have found that the lack of awareness about COVID-19 among the public was the most common obstacle that undermines all efforts to control the outbreak [12]. Accordingly, in this study, we tried to investigate the prevalence of COVID-19 misinformation among the Yemeni general population and its association with vaccine acceptance and perception. Also, factors affecting the perceptions, misinformation, and willingness to vaccinate were evaluated.

## Methods

### Study design and settings

This study is a cross-sectional online survey in Yemen. Subject recruitment and data collection were conducted online using the snowball sampling technique. Data were collected in the first two weeks of the outbreak in Yemen from April 12, 2020, to April 26, from four major cities in Yemen: Sana’a, Al-Hudaidah, Ta’aiz, and Aden. In each city, the response was from both urban and rural households. All included subjects were above 18 years. Social media platforms like WhatsApp and Facebook were used to distribute google form questionnaires. Subjects were recruited in the study using the simplified snowball sampling technique, and they were requested to pass the invitation to their contacts; the estimated time to complete the survey was around 10 minutes.

### Ethical approval

This study was part of a project about COVID-19 in Yemen. The current study was approved by the ethical committee of the Medical Research, University of Science and Technology, Sana’a, Yemen, with the following approval number: ECA/UST189. An electronic informed consent statement to be ticked by all participants who agreed to participate, those who did not tick it will not be able to fill the questionnaire.

### Data collection tool

The constructed questionnaire was divided into four sections; section A: demographics data such as age, gender, marital status, residential area, medical insurance, education level, work nature, presence of chronic disease, smoking, and khat chewing status (S1 File). Section B: misinformation about COVID-19. This section contains seven statements with five possible answers on a 5-Likert scale (strongly disagree to agree strongly) about commonly spread misinformation at the time of data collection. The scores for each statement ranged from 1 to 5, and the overall score ranged from 7 to 35, a higher score, indicated a higher level of misinformation. For inferential analysis, misinformation was categorized using the median as misinformed (>19) and informed (≤19). Section C: COVID-19 perception, which was divided into three subsections; perceived susceptibility, perceived severity/threat, and perceived worry. Perceived susceptibility is the individual’s belief of the chances to get a COVID-19. It contained four items with four possible choices (not at all likely, slightly likely, somewhat likely, and very likely), and the total score ranged from 4 to 16. For perceived severity, belief about how serious or dangerous the COVID-19 and its consequences are, a 4-Likert scale ranged from not dangerous at all to very dangerous was used, and the total score ranged from 5 to 20. The perceived worry (5 items) was assessed on a 4-Likert scale (not at all worried, slightly worried, moderately worried, and very worried), and the total score ranged from 5 to 20. The final score for perception subscales was categorized into perceived and not perceived using the median for Chi-Square analysis.

Section D contained two questions, with yes, no, not sure answers, focused on the vaccine acceptance among Yemeni people in two situations, a free effective vaccine and a vaccine with a cost of about 10 thousand Yemeni Rials (approximately 15$ at the time of study period). The constructed questionnaire was structurally designed to be based on self-reporting, and some of its components were adapted from previously published studies about the Ebola virus [13, 14].

The questionnaire’s contents were checked, evaluated, and validated by a panel of experts with academic, clinical, and questionnaire construction backgrounds. The questionnaire was checked for reliability using Cronbach’s Alpha. The results from Cronbach’s Alpha were as following; COVID-19 misinformation (0.647), perceived susceptibility (0.870), perceived severity (0.757), and perceived worry (0.830).

### Sample size

A sample size of 385 was estimated using the Daniel formula [15] with an expected prevalence of 50% for misinformation and vaccination acceptance, to have the highest number of respondents [16], using a confidence interval of 95%, precision of 0.05 and estimated population of 30 million in Yemen [17]. We recruited additional 100 subjects to reach a final sample of 484 and get at least 80% power.

### Statistical analysis

Descriptive statistics were used to analyze the sociodemographic characteristics and the responses to questions concerning misinformation, perceptions, and acceptance of the COVID-19’s vaccines. Frequencies and percentages were used to present categorical variables. A Pearson Chi-Square was conducted to detect the association between respondents’ characteristics and their misinformation, perception, and vaccination acceptance. Statistical analysis was done using the Statistical Package for the Social Sciences (SPSS) (version 27.0; IBM corp). P<0.05 was taken as a cut point for statistically significant results.

## Results

The total number of subjects who participated in this study was 484. The median (interquartile range) age was 31.5 (14). Approximately 61% of whom were males and had a university education. While more than half of the participants were khat chewers, only 18% were smokers. Also, the majority of participants (85%) were free from chronic diseases. (Table 1).

**Table 1:** Socio-demographic characteristics (n = 484).

Parameter	Number	Percentage
**Gender**		
Male	295	61
Female	189	39
**Age**		
31 and less	242	50
More than 31	242	50
**Resident area**		
Rural	63	13
Urban	421	87
**Medical insurance**		
Yes	76	15.7
No	408	84.3
**Marital status**		
Married	315	65.1
Single	157	32.4
Divorced/widow	12	2.5
**Educational level**		
Non-university	180	37.2
University	304	62.8
**Current work nature**		
Unemployed	156	32.2
Employed	194	40.1
Daily wage jobs	134	27.7
**Income**		
<100$	247	51
≥100%	237	49
**Current smoker**		
Yes	89	18.4
No	395	81.6
**Chronic diseases**		
Yes	71	14.7
No	413	85.3
**Current khat chewers**		
Yes	245	50.6
No	239	49.4

### Misinformation

For the prevalence of misinformation, slightly less than a quarter of participants (23.2%) agree or strongly agree that COVID-19 was a human-made virus designed by pharmaceutical companies for financial gains, while almost two fifths (40%) saw the virus as a human-made biological weapon (Table 2). The proportion of respondents who agreed or strongly agreed most people who are infected with coronavirus would die just under a third (31%). We found that men (p = 0.01), university education (p<0.001), higher income (p<0.001), being employed (p<0.001), and living in urban areas (p = 0.016) are significantly associated with being informed (lower level of misinformation) (Table 4). The overall median for misinformation (interquartile range) was 19 (6).

**Table 2:** Covid-19 misinformation (n = 484).

Item	Strongly disagree	Disagree	Neutral	Agree	Strongly agree
COVID-19 is human-made for pharmaceutical companies’ financial gains	68 (14)	100 (20.7)	204 (42.1)	71 (14.7)	41 (8.5)
COVID-19 was created by human as a biological weapon	43 (8.9)	64 (13.2)	189 (39)	134 (27.7)	54 (11.2)
COVID-19 virus cannot be transmitted in areas with hot climates	41 (8.5)	125 (25.8)	152 (31.4)	154 (31.8)	12 (2.5)
Children will not be infected or carry the virus	105 (21.7)	207 (42.8)	98 (20.2)	68 (14.0)	6 (1.2)
Most people who get the coronavirus will die	90 (18.6)	179 (37.0)	61 (12.6)	126 (26.0)	28 (5.8)
COVID-19 can be prevented or treated by eating raw garlic and drinking hot tea containing anise	49 (10.1)	129 (26.7)	160 (33.1)	132 (27.3)	14 (2.9)
Antibiotics are effective in preventing and treating the new coronavirus	145 (30.0)	147 (30.4)	149 (30.8)	41 (8.5)	2 (0.4)

### Willingness to vaccinate

**Fig 1:**
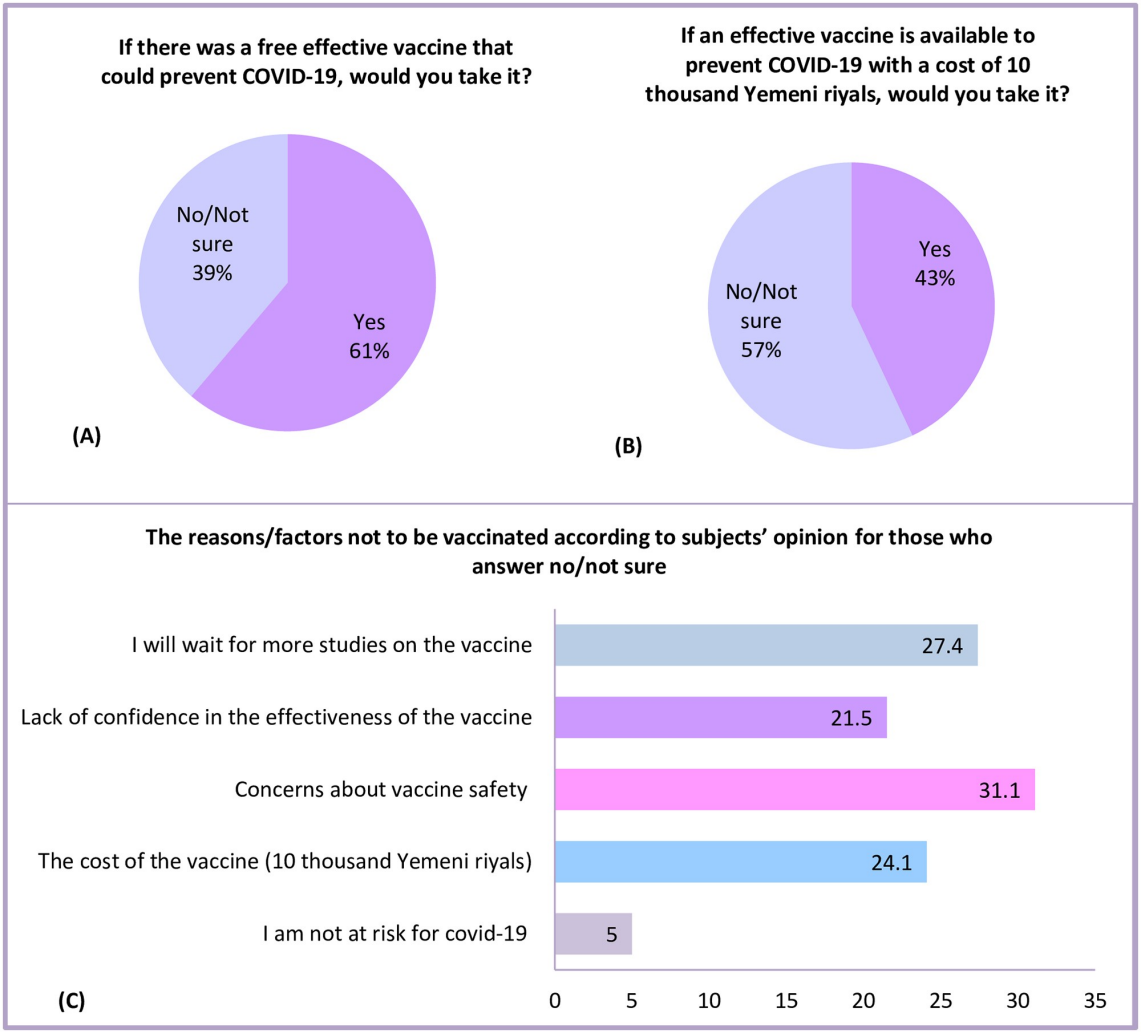
A-C Willingness to vaccinate and the barriers that are hindering Yemeni people from vaccination (n = 484).

Around two-fifths of respondents (61%) would take the vaccine if they were offered a free one (Fig 1A). However, the vaccination acceptance decreased to 43% if they had to purchase it (Fig 1B). Men were more likely to accept the vaccine than women, whether provided for free or to purchase it (Free: 66.4% vs 52.9%; respectively, (p = 0.003), purchase: 47.5% vs 36%; respectively, (p = 0.013) (Table 4). Also, respondents who were above 31 years of age were more willing to be vaccinated for free than the younger group (69.4% vs 52.9%; respectively, p <0.001). Moreover, employed individuals were more willing to purchase a vaccine than unemployed and daily wage workers (49.5%, 36.5%, vs 41%; respectively, (p = 0.04) (Table 4). Notably, respondents with lower monthly income (less than 100$) were more likely to reject the vaccination whether provided for free (48.6% vs 28.7%; respectively, p < 0.001) or with a cost of 15$ (70% vs 43.5%; respectively, p < 0.001).

### Perceived susceptibility

The overall median for perceived susceptibility (interquartile range) was 8 (5). Only 20.9% of respondents believed it was somewhat likely or very likely that they would be infected with COVID-19 (Table 3). Also, less than one-fifth of participants believed it was somewhat likely or very likely that their family would get a COVID-19 infection. Interestingly, when asked about their city and governorate, the perceived susceptibility increased to 35.4% and 42.6%, respectively. Statistically significant associations between university education (p<0.001), living in the urban areas (p = 0.003), and being employed (p = 0.003) with perceived susceptibility were observed in Table 4.

**Table 3:** Perception of the community towards COVID-19 (n = 484).

**Perceived susceptibility**	**Not at All Likely**	**Slightly Likely**	**Somewhat Likely**	**Very Likely**
How likely is it that you will be infected with COVID-19 within the next few months?	178 (36.8)	205 (42.4)	76 (15.7)	25 (5.2)
How likely is it that one of your family will be infected with COVID-19 within the next few months?	177 (36.6)	216 (44.6)	68 (14.0)	23 (4.8)
How likely is the onset of the COVID-19 outbreak in your city within the next few months?	95 (19.6)	218 (45.0)	87 (18.0)	84 (17.4)
How likely is the onset of the COVID-19 outbreak in your governorate within the next few months?	65 (13.4)	213 (44.0)	100 (20.7)	106 (21.9)
**Perceived severity/threat**	**Not dangerous at all**	**Slightly dangerous**	**Moderately dangerous**	**Very dangerous**
In general, how dangerous do you think the COVID-19 pandemic is?	5 (1.0)	34 (7.0)	108 (22.3)	337 (69.6)
How dangerous do you think it would be if you were diagnosed with COVID-19?	7 (1.4)	47 (9.7)	110 (22.7)	320 (66.1)
How dangerous do you think would COVID-19 be on you if it began to spread to your community?	32 (6.6)	99 (20.5)	156 (32.2)	197 (40.7)
How dangerous do you think it would be for your city if COVID-19 started spreading in your governorate?	10 (2.1)	21 (4.3)	66 (13.6)	387 (80.0)
How dangerous do you think the consequences of COVID-19 disease would be on your country?	8 (1.7)	13 (2.7)	58 (12.0)	405 (83.7)
**Perceived Worry**	**Not at all worried**	**Slightly worried**	**Moderately worried**	**Very worried**
How worried are you about COVID-19 at this moment?	69 (14.3)	211 (43.6)	93 (19.2)	111 (22.9)
How worried are you that you will be infected with COVID-19 in the next few months?	96 (19.8)	223 (46.1)	72 (14.9)	93 (19.2)
How worried are you that someone you know (relatives, friends, etc.) will be infected with COVID-19 in the next few months?	39 (8.1)	166 (33.3)	99 (20.5)	185 (38.2)
How worried are you that an outbreak of COVID-19 will happen in your city?	13 (2.7)	99 (20.5)	79 (16.3)	293 (60.5)
How worried are you that you will not be able to go outside of your house if a COVID-19 outbreak happened in your city?	73 (15.1)	121 (25.0)	84 (17.4)	206 (42.6)

**Table 4:** Association between demographics and subjects’ perception, vaccine acceptance, and misinformation (n = 484).

Parameter	Susceptibility (Perceived, N = 223)	P-value^a^	Severity (Perceived, N = 234)	P-value^a^	Worry (Perceived, N = 216)	P-value^a^	Free Vaccine (Accepted, N = 296)	P-value^a^	Purchased vaccine (Welling, N = 208)	P-value^a^	Misinformation (Informed, N = 253)	P-value^a^
**Gender**		0.26		0.29		0.62		0.003		0.013		0.01
Male	142(51.9)		137(51.3)		129(46)		196(66.4)		140(47.5)		168(56.9)	
Female	81(57.1)		97(46.4)		87(43.7)		100(52.9)		68(36)		85(45)	
**Age**		0.083		0.045		0.067		<0.001		0.359		0.17
Less than 31	121(54.3)		128(43.8)		98(48.8)		168(52.9)		109(40.9)		134(49.2)	
More than 31	102(45.7)		106(52.9)		118(40.5)		128(69.4)		99(45)		119(55.4)	
**Education**		<0.001		0.024		0.044		0.241		0.284		<0.001
University	159(52.3)		135(57.7)		125(50.6)		193(63.2)		125(41.1)		178(61.5)	
Non-university	64(35.6)		99(42.3)		91(41.1)		104(57.8)		83(46.1)		66(36.7)	
**Residential area**		0.003		0.000		0.609		0.336		0.179		0.016
Urban	205(48.7)		189(44.9)		186(44.2)		254(60.3)		176(41.8)		229(54.4)	
Rural	18(28.6)		45(71.4)		30(47.6)		42(66.7)		32(50.8)		24(38.1)	
**Medical insurance**		0.134		0.092		0.818		0.894		0.178		0.187
Yes	41(53.9)		30(39.5)		33(43.4)		47(61.8)		38(50)		45(59.2)	
No	182(44.6)		204(50)		183(49.9)		249(61)		170(41.7)		208(51)	
**Work**		0.003		0.064		0.754		0.060		0.045		<0.001
Employed	104(53.6)		85(43.8)		83(42.8)		131(67.5)		96(49.5)		124(63.9)	
Unemployed	73(46.8)		73(46.8)		73(46.8)		90(57.7)		57(36.5)		74(47.4)	
Daily Wages	46(34.3)		76(56.7)		60(44.8)		75(52)		55(41)		55(41)	
**Income**		0.381		0.035		0.216		<0.001		<0.001		<0.001
<100$	109(44.1)		131(53)		117(47.4)		127(51.4)		74(30)		94(38.1)	
≥100$	114(48.1)		103(43.5)		99(41.8)		169(71.3)		134(56.5)		159(67.1)	
**Smoking**		0.347		0.644		0.521		0.891		0.374		0.125
Yes	45(50.6)		45(50.6)		37(41.6)		55(61.8)		42(47.2)		40(54.9)	
No	178(45.1)		191(47.8)		179(45.3)		241(61)		166(42)		213(53.9)	
**Khat chewing**		0.554		0.170		0.669		0.023		0.049		0.85
Yes	117(47.8)		126(51.4)		109(43.7)		162(66.1)		116(47.3)		127(51.8)	
No	106(44.4)		108(45.2)		105(45.6)		134(56.1)		92(38.5)		126(52.7)	
**Chronic conditions**		0.659		0.001		0.008		0.141		0.699		0.29
Yes	31(43.7)		47(66.2)		42(59.2)		49(69)		32(45.5)		33(46.5)	
No	192(46.5)		184(45.3)		174(42.1)		247(59.8)		176(42.6)		220(53.3)	

^a^ P-values were calculated using Chi-Square test.

### Perceived severity/threats

The overall median for perceived severity (interquartile range) was 18 (3). The perceived threat data indicated that around 70% of respondents thought of COVID-19 as a dangerous disease. However, only two-fifths perceived COVID-19 as very dangerous to them if it started spreading in their community. Remarkably, the perceived severity increased among the respondents when they were asked about how dangerous COVID-19 would be on their city, and its consequences would be on the country, with 80% and 83% saw it as very dangerous, respectively. Respondents who were above 31 years of age, had low income (<100$), and those who were living in the urban area or with a chronic disease condition are more likely to have a perception of the disease’s severity (Table 4).

### Perceived worry

The overall median for perceived worry (interquartile range) was 14 (6). More than half of the respondents (57.9%) were not worried at all or slightly worried about the COVID-19 situation. Moreover, just over a third of subjects (34.1%) were at least moderately worried about attracting the infection themselves in the next few months. This percentage of perceived worry increased to more than half (58.7%) when respondents were asked about their relatives and friends. While the majority of people (76.8%) were moderately worried or very worried that an outbreak would happen in their cities, less percentage of them (58%) were worried about the restrictions that might come with a sudden outbreak, like being unable to go out. Perceived worry was significantly associated with respondents who had a university education (p = 0.04) or had a chronic disease condition (p = 0.008) (Table 4).

#### The effect of misinformation on perceptions and willingness to vaccinate

The data showed that respondents who believed that pharmaceutical companies made the virus for financial gains had a significantly lower acceptance rate for a free vaccine (50.9% vs 64.2%; respectively, (p = 0.011) or with a cost of 15$ (34.8% vs 45.5%; respectively, (p = 0.046) compared to those who did not think so (Table 5). The participants who believed that most people with COVID-19 would die had significantly higher perception for severity ((p = 0.000) and worry for the disease (p = 0.000). Interestingly, people who thought that COVID-19 could be prevented by eating garlic had a significantly lower level of susceptibility perception for COVID-19 (p = 0.04).

**Table 5:** Association between subjects’ misinformation, perception and vaccine acceptance (n = 484).

Misinformation	Susceptibility (Perceived, N = 223)	P-value^a^	Severity (Perceived, N = 234)	P-value^a^	Worry (Perceived N = 216)	P-value^a^	Free vaccine (Accepted, N = 296)	P-value^a^	Purchase the vaccine (Welling, N = 208)	P-value^a^
**COVID-19 is human-made for pharmaceutical companies’ financial gains**		0.463		0.972		0.662		0.011		0.046
Strongly agree/agree	55(49.1)		54(48.2)		52(46.4)		57(50.9)		39(34.8)	
Strongly disagree/disagree/neutral	168(45.2)		180(48.4)		164(41.1)		239(64.2)		169(45.5)	
**COVID-19 was created by human as a biological weapon**		0.387		0.836		0.586		0.253		0.275
Strongly agree/agree	82(43.6)		92(48.9)		81(43.1)		109(58)		75(39.9)	
Strongly disagree/disagree/neutral	141(47.6)		142(48)		135(45.6)		187(63.2)		133(44.9)	
**COVID-19 virus cannot be transmitted in areas with hot climates**		0.068		0.738		0.835		0.620		0.795
Strongly agree/agree	67(40.4)		82(49.4)		73(44)		99(59.6)		70(42.2)	
Strongly disagree/disagree/neutral	156(49.1)		152(47.8)		143(45)		197(61.9)		138(43.4)	
**Children will not be infected by the virus**		0.596		0.844		0.306		0.399		0.646
Strongly agree/agree	74(43.2)		35(47.3)		29(39.2)		42(56.8)		30(45.5)	
Strongly disagree/disagree/neutral	191(46.6)		199(48.5)		187(45.6)		254(62)		178(43.4)	
**Most people who get the coronavirus will die**		0.838		<0.001		<0.001		0.813		0.971
Strongly agree/agree	72(46.8)		93(60.4)		88(57.1)		93(60.4)		66(42.9)	
Strongly disagree/disagree/neutral	151(45.8)		141(42.7)		128(38.8)		203(61.5)		142(43)	
**COVID-19 can be prevented by eating garlic and drinking tea containing anise**		0.041		0.935		0.818		0.642		0.882
Strongly agree/agree	57(39)		71(48.6)		64(43.8)		87(59.6)		62(42.5)	
Strongly disagree/disagree/neutral	166(49.1)		163(48.2)		152(45)		209(61.8)		146(43.2)	
**Antibiotics are effective in preventing and treating the virus**		0.222		0.946		0.221		0.577		0.624
Strongly agree/agree	16(37.2)		21(48.8)		23(53.5)		28(65.1)		20(46.5)	
Strongly disagree/disagree/neutral	207(46.9)		213(48.3)		193(43.8)		268(60.8)		188(42.6)	

^a^ P-values were calculated using Chi-Square test.

## Discussion

Overall, misinformation was not high among Yemenis except for the misconception that humans have created COVID-19 as a biological weapon, where approximately two-fifths believed so. This was consistent with a recent finding from Nigeria, where 39% believed that COVID-19 was part of biological warfare [18]. A higher percentage (57%) of the population was reported to have the same belief in Jordan [19]. Even though experts refuted the idea that COVID-19 was engineered in the laboratories, these findings suggest that this misconception is still common.

Female gender, non-university education, low-income, and rural areas were significantly associated with being misinformed about COVID-19. These results align with those reported in Jordan, where beliefs that COVID-19 is part of a conspiracy theory and biological warfare were more common in females and among people with low education levels and income [19]. Such findings could be attributed to multiple factors; for example, in Yemen, males have better access to education than females, reports from UNESCO have shown that in Yemen, the literacy among male subjects was more than 30% higher than the female, with around 85% of male subjects reaching high school [20]. Also, Rampersad and Althyabi found that gender can weakly and indirectly influence misinformation acceptance, while education showed a strong negative effect on accepting rumors and misinformation [21]. Well-educated people are less likely to accept misinformation. They depend on reliable sources and tend to search for expert opinions rather than accept misinformation, and usually, they have more analytical thinking [22]. The lower level of misinformation among urban area residents could be partially explained by the availability of better access to education and health facilities than those in rural areas due to higher population density, closer proximity, and transportation availability [23].

We found that 61% of the public in Yemen agreed to be vaccinated against COVID-19. This finding was lower than that reported from an international survey of 19 countries which found 71.5% of participants were willing to take the vaccine [24]. Also, a higher percentage of vaccine acceptance was reported in France (75%) [25]. Similarly, an online survey done in the United Kingdom prior to any vaccine licensure found that only a minority of respondents (11.7%) were strongly hesitant to be vaccinated [26]. The main barriers that made our participants reluctant to be vaccinated were concerns over vaccine safety, efficacy, and price. The price, which is 15 USD only, decreased the acceptance rate by 20%, suggesting that cost is a significant barrier to vaccination in Yemen, and a higher rejection rate would be expected if people in Yemen had to purchase a higher-cost vaccine. This is because Yemen is one of the Middle East’s poorest countries, where almost 50% of the population lives below the poverty line, and around 20% earn only 1.2 USD per day [27, 28]. Also, the association between low financial status and vaccination rejection was apparent in this study; those who had a monthly income of less than 100 USD and unemployed individuals were less likely to purchase a vaccine. Notably, the acceptance rate for a free COVID-19 vaccine was also suboptimal (61%) and below the percentage needed to meet the anticipated levels of herd immunity, suggesting that it is vital for healthcare authorities and international organizations working in Yemen not only to ensure that these populations have a free vaccine, but also that trust in the vaccine efficacy and safety is built up prior to roll-out vaccination campaigns. In addition, more efforts should be made to educate females, younger, and low-income groups about the vaccine, as these were more likely to reject a free vaccination, in alignment with previous studies from the UK and France [6, 25, 26, 29, 30].

Another clear finding from this study is that misinformation could influence people’s decision to get vaccinated. In this light, people who believed that COVID-19 is human-made for pharmaceutical companies’ financial gains were more likely to reject the vaccine. Such finding is concordant to other emerging studies in the context of COVID-19 that have linked specific theories of conspiracy to lower willingness to adopt behaviors in public health. Brennen et al. found that much of the misleading information about COVID-19, like the conspiracy theory is originated from fake news, which can be associated with photoshopped pictures usually used as fake pieces of evidence and can be spread easily through social media [31]. This kind of conspiracy beliefs can present a significant obstacle for mass vaccination programs, and it can play a role in facilitating the virus spread because those who believe in such theories tend not to take preventive measures like social distancing and do not follow standard operating procedures [32, 33]. Therefore, it is important to debunk this kind of theory and try to confront the public’s misinformation to control the virus spread and increase vaccine acceptance among the Yemeni population.

For perceptions, the participants showed a low self and family perception regarding the susceptibility of being infected. Similarly, during the H5N1 virus outbreak in Hong Kong, it was found that the vast majority of subjects had less perception toward the risk of being infected themselves compared to the risk of a general outbreak in their cities [34]. Furthermore, people were too concerned and worried about their cities and communities in case of a major outbreak, which is harmonious with our findings. Factors such as age, employment, urban areas, and education were also associated with a better perception among the included subjects. In the United States, they have found that the perception toward the risks of COVID-19 was higher among older subjects, while the younger subjects were more worried about the pandemic. Furthermore, women’s perception of risks was slightly higher than men, and they were more worried than men; however, the interactions between gender and age were insignificant [35]. Interestingly, our participants’ level of perception increased (perceived susceptibility, severity, and worry) when asked about themselves, their relatives, and cities, respectively, indicating they had a higher perceived susceptibility and worry for their relatives and communities. This suggests that altruistic messaging to protect their families, friends, country might be a useful strategy among the Yemenis to increase their acceptance rate for the COVID-19 vaccine [33].

### Strength and limitations of the study

There are a few limitations to the conducted study. Responses were received mainly from four major cities in Yemen, limiting the generalization of findings to the whole country. Another limitation was related to the use of social networks and snowballing sampling technique to recruit participants. Many people in Yemen do not have electricity, electronic devices or access to social networks; thus, the survey did not capture their perceptions and vaccine acceptance. Therefore, this may contribute to selection bias and decrease the findings’ representativeness. A more systematic and comprehensive sampling procedure is required to improve the generalizability. Also, due to the study design (cross-sectional), the results represent only the point when the data has been collected. As the data collection was done before any awareness vaccination campaigns, the public’s perceptions and vaccination acceptance might have changed over time. Therefore, future post awareness campaign studies are warranted.

Despite the presented limitations, the sample size was adequate, and the participants were from the largest cities in Yemen, which gives a close enough image and a realistic idea about the presented topic in Yemen at the first two weeks of the outbreak in Yemen. The conducted study provides a good insight into the misinformation, perceptions, and acceptance of the vaccine among Yemenis, opening the door for more comparative research and investigations to be conducted in the future or after awareness campaigns and educational interventions. The study also provides a good insight into COVID-19 vaccine acceptance in a low-income, less-developed country like Yemen. Importantly, the study findings provide useful insight for policymakers, healthcare planners, and international organizations planning to support or donate vaccines to Yemen.

## Conclusion

The study revealed that the acceptance rate to take a vaccine was suboptimal and significantly affected by gender, misinformation, cost, and income. Furthermore, being female, non-university educated, with low income, and living in rural areas were associated with higher susceptibility to misinformation about COVID-19. These findings show a clear link between misinformation susceptibility and willingness to vaccinate. Therefore, focused awareness campaigns to decrease misinformation and emphasize the vaccination’s safety and efficacy might be fundamental before initiating any vaccination program in Yemen.

## Supporting information

S1 File Study questionnaire. (PDF)

S2 File Study dataset. (SAV)

## References

[1] Mullard A. How COVID vaccines are being divvied up around the world. Nature. 2020 [cited 16 Dec 2020]. doi: 10.1038/d41586-020-03370-6 33257891

[2] Status of COVID-19 Vaccines within WHO EUL/PQ evaluation process. Guidance document by WHO; 2021.

[3] Zawiah M, Al-Ashwal FY, Saeed RM, Kubas M, Saeed S, Khan AH, et al. Assessment of Healthcare System Capabilities and Preparedness in Yemen to Confront the Novel Coronavirus 2019 (COVID-19) Outbreak: A Perspective of Healthcare Workers. Front Public Health. 2020;8: 419. doi: 10.3389/fpubh.2020.00419 32850608 PMC7399068

[4] Nasser A, Zakham F. A strategy for SARS-CoV-2 vaccination in Yemen. The Lancet. 2021;397: 2247. doi: 10.1016/S0140-6736(21)01016-3 34119057 PMC8192093

[5] INGO briefing for the UNGA: The humanitarian situation in Yemen after seven years of conflict (September 2021)—Yemen. In: ReliefWeb [Internet]. [cited 15 Sep 2021]. https://reliefweb.int/report/yemen/ingo-briefing-unga-humanitarian-situation-yemen-after-seven-years-conflict-september

[6] Sherman SM, Smith LE, Sim J, Amlôt R, Cutts M, Dasch H, et al. COVID-19 vaccination intention in the UK: results from the COVID-19 vaccination acceptability study (CoVAccS), a nationally representative cross-sectional survey. Hum Vaccines Immunother. 2020;0: 1–10. doi: 10.1080/21645515.2020.1846397 33242386 PMC8115754

[7] Mohamed NA, Solehan HM, Rani MDM, Ithnin M, Isahak CIC. Knowledge, acceptance and perception on COVID-19 vaccine among Malaysians: A web-based survey. PLOS ONE. 2021;16: e0256110. doi: 10.1371/journal.pone.0256110 34388202 PMC8362951

[8] Ranjit YS, Shin H, First JM, Houston JB. COVID-19 protective model: the role of threat perceptions and informational cues in influencing behavior. J Risk Res. 2021;24: 449–465. doi: 10.1080/13669877.2021.1887328

[9] Verger P, Dubé E. Restoring confidence in vaccines in the COVID-19 era. Expert Rev Vaccines. 2020;0: 1–3. doi: 10.1080/14760584.2020.1825945 32940574

[10] Al-Mohaithef M, Padhi BK. Determinants of COVID-19 Vaccine Acceptance in Saudi Arabia: A Web-Based National Survey. J Multidiscip Healthc. 2020;13: 1657–1663. doi: 10.2147/JMDH.S276771 33262600 PMC7686470

[11] Kreps S, Prasad S, Brownstein JS, Hswen Y, Garibaldi BT, Zhang B, et al. Factors Associated With US Adults’ Likelihood of Accepting COVID-19 Vaccination. JAMA Netw Open. 2020;3: e2025594–e2025594. doi: 10.1001/jamanetworkopen.2020.25594 33079199 PMC7576409

[12] Al-Ashwal FY, Kubas M, Zawiah M, Bitar AN, Saeed RM, Sulaiman SAS, et al. Healthcare workers’ knowledge, preparedness, counselling practices, and perceived barriers to confront COVID-19: A cross-sectional study from a war-torn country, Yemen. PLOS ONE. 2020;15: e0243962. doi: 10.1371/journal.pone.0243962 33306750 PMC7732096

[13] Schol LGC, Mollers M, Swaan CM, Beaujean DJMA, Wong A, Timen A. Knowledge, perceptions and media use of the Dutch general public and healthcare workers regarding Ebola, 2014. BMC Infect Dis. 2018;18: 18. doi: 10.1186/s12879-017-2906-7 29310571 PMC5759181

[14] Kelly B, Squiers L, Bann C, Stine A, Hansen H, Lynch M. Perceptions and plans for prevention of Ebola: results from a national survey. BMC Public Health. 2015;15: 1136. doi: 10.1186/s12889-015-2441-7 26572610 PMC4647489

[15] Daniel WW, Cross CL. Biostatistics: A Foundation for Analysis in the Health Sciences. John Wiley & Sons; 2018.

[16] Naing L, Winn T, Rusli BN. Practical issues in calculating the sample size for prevalence studies. Arch Orofac Sci. 2006;1. Available: http://mymedr.afpm.org.my/publications/42468

[17] Yemen Population (2020)—Worldometer. [cited 19 Dec 2020]. https://www.worldometers.info/world-population/yemen-population/

[18] Olatunji OS, Ayandele O, Ashirudeen D, Olaniru OS. “Infodemic” in a pandemic: COVID-19 conspiracy theories in an african country. Soc Health Behav. 2020;3: 152. doi: 10.4103/SHB.SHB_43_20

[19] Sallam M, Dababseh D, Yaseen A, Al-Haidar A, Taim D, Eid H, et al. COVID-19 misinformation: Mere harmless delusions or much more? A knowledge and attitude cross-sectional study among the general public residing in Jordan. PLOS ONE. 2020;15: e0243264. doi: 10.1371/journal.pone.0243264 33270783 PMC7714217

[20] Yemen. 27 Nov 2016 [cited 19 Feb 2021]. http://uis.unesco.org/en/country/ye

[21] Rampersad G, Althiyabi T. Fake news: Acceptance by demographics and culture on social media. J Inf Technol Polit. 2020;17: 1–11. doi: 10.1080/19331681.2019.1686676

[22] Sharon AJ, Baram‐Tsabari A. Can science literacy help individuals identify misinformation in everyday life? Sci Educ. 2020;104: 873–894. 10.1002/sce.21581

[23] Owens A. Income Segregation between School Districts and Inequality in Students’ Achievement. Sociol Educ. 2018;91: 1–27. doi: 10.1177/0038040717741180

[24] Lazarus JV, Ratzan SC, Palayew A, Gostin LO, Larson HJ, Rabin K, et al. A global survey of potential acceptance of a COVID-19 vaccine. Nat Med. 2020; 1–4. doi: 10.1038/s41591-019-0740-8 33082575 PMC7573523

[25] Detoc M, Bruel S, Frappe P, Tardy B, Botelho-Nevers E, Gagneux-Brunon A. Intention to participate in a COVID-19 vaccine clinical trial and to get vaccinated against COVID-19 in France during the pandemic. Vaccine. 2020;38: 7002–7006. doi: 10.1016/j.vaccine.2020.09.041 32988688 PMC7498238

[26] Freeman D, Loe BS, Chadwick A, Vaccari C, Waite F, Rosebrock L, et al. COVID-19 vaccine hesitancy in the UK: the Oxford coronavirus explanations, attitudes, and narratives survey (Oceans) II. Psychol Med. undefined/ed; 1–15. doi: 10.1017/S0033291720005188 33305716 PMC7804077

[27] Economy of Yemen. In: Fanack.com [Internet]. [cited 9 Feb 2021]. https://fanack.com/yemen/economy/

[28] Qirbi N, Ismail SA. Health system functionality in a low-income country in the midst of conflict: the case of Yemen. Health Policy Plan. 2017;32: 911–922. doi: 10.1093/heapol/czx031 28402469

[29] McAndrew S, Allington D. Mode and Frequency of Covid-19 Information Updates, Political Values, and Future Covid-19 Vaccine Attitudes. PsyArXiv; 2020. doi: 10.31234/osf.io/j7srx

[30] Murphy J, Vallières F, Bentall RP, Shevlin M, McBride O, Hartman TK, et al. Preparing for a COVID-19 vaccine: Identifying and psychologically profiling those who are vaccine hesitant or resistant in two general population samples. PsyArXiv; 2020. doi: 10.31234/osf.io/pev2b

[31] Brennen JS, Simon FM, Nielsen RK. Beyond (Mis)Representation: Visuals in COVID-19 Misinformation. Int J Press. 2021;26: 277–299. doi: 10.1177/1940161220964780 PMC7548543 38603033

[32] Romer D, Jamieson KH. Conspiracy theories as barriers to controlling the spread of COVID-19 in the U.S. Soc Sci Med. 2020;263: 113356. doi: 10.1016/j.socscimed.2020.113356 32967786 PMC7502362

[33] Loomba S, de Figueiredo A, Piatek SJ, de Graaf K, Larson HJ. Measuring the impact of COVID-19 vaccine misinformation on vaccination intent in the UK and USA. Nat Hum Behav. 2021; 1–12. doi: 10.1038/s41562-021-01049-0 33547453

[34] Lau JT, Kim JH, Tsui HY, Griffiths S. Anticipated and current preventive behaviors in response to an anticipated human-to-human H5N1 epidemic in the Hong Kong Chinese general population. BMC Infect Dis. 2007;7: 18. doi: 10.1186/1471-2334-7-18 17359545 PMC1845150

[35] Barber SJ, Kim H. COVID-19 Worries and Behavior Changes in Older and Younger Men and Women. J Gerontol Ser B. 2021;76: e17–e23. doi: 10.1093/geronb/gbaa068 32427341 PMC7313781

