# Supplementary material for: Misinformation, perceptions towards COVID-19 and willingness to be vaccinated: A population-based survey in Yemen
Source: PLoS One. 2021 Oct 29;16(10):e0248325. doi: 10.1371/journal.pone.0248325 (PMC8555792; doi:10.1371/journal.pone.0248325)
Supplement: S1 File — (PDF) [file pone.0248325.s001.pdf]

Misinformation, perceptions towards the COVID-19, and willingness to vaccinate in Yemen: A population-based survey

Section A: Demographic data

-**Your Age**: …………….

-**Your Gender**: Male Female

-**Current place of residence (governorate)**: ……………….

- **Residence area**: Urban Rural

- **Monthly income**:

 Less than 30 thousand Yemeni Rials 30 to less than 60 thousand Yemeni Rials

 60 thousand to less than 120 thousand 120 to less than 180 thousand Yemeni Rials

 180 thousand to less than 240 thousand Equal or more than 240 thousand

- **Marital status:** Single Married Divorced/widowed

-**Do you have medical insurance?**

 Yes No

-**Education level:**

 No formal education

 Primary

 Secondary

 Diploma

 Bachelor

 Postgraduate

-**Your current work nature**: Unemployed Employed Daily-wage workers

- Are you a current smoker? Yes No

- Are you a current Kat chewer? Yes No

- Do you have any chronic diseases (such as high blood pressure, diabetes, heart disease, lung disease, liver disease, etc.)? Yes No

**Section B: COVID-19 Misinformation:**<br>In this section, we want to know how much you agree or disagree with the following sentences						
		**Strongly disagree**	**Disagree**	**Neutral**	**Agree**	**Strongly agree**
1	COVID-19 is human-made for pharmaceutical companies' financial gains					
2	COVID-19 was created by humans as a biological weapon					
3	COVID-19 virus cannot be transmitted in areas with hot climates					
4	Children will not be infected or carry the virus					
5	Most people who get the coronavirus will die					
6	COVID-19 can be prevented or treated by eating raw garlic and drinking hot tea containing anise					
7	Antibiotics are effective in preventing and treating the new coronavirus					

**Section C: COVID-19 perceptions**					
**A. Perceived susceptibility**		**Not at All Likely**	**Slightly Likely**	**Somewhat Likely**	**Very Likely**
**1**	How likely is it that you will be infected with COVID-19 within the next few months?				
**2**	How likely is it that one of your family will be infected with COVID-19 within the next few months?				
**3**	How likely is the onset of the COVID-19 outbreak in your city within the next few months?				

**4**	How likely is the onset of the COVID-19 outbreak in your governorate within the next few months?				
**B.**	**Perceived severity/threat**	**Not dangerous at all**	**Slightly dangerous**	**Moderately dangerous**	**Very dangerous**
**1**	In general, how dangerous do you think the COVID-19 pandemic is?				
**2**	How dangerous do you think it would be if you were diagnosed with COVID-19?				
**3**	How dangerous do you think would COVID-19 be on you if it began to spread to your community?				
**4**	How dangerous do you think it would be for your city if COVID-19 started spreading in your governorate?				
**5**	How dangerous do you think the consequences of COVID-19 disease would be on your country?				
**C.**	**Perceived Worry**	**Not at all worried**	**Slightly worried**	**Moderately worried**	**Very worried**
**1**	How worried are you about COVID-19 at this moment?				
**2**	How worried are you that you will be infected with COVID-19 in the next few months?				
**3**	How worried are you that someone you know (relatives, friends, etc.) will be infected with COVID-19 in the next few months?				
**4**	How worried are you that an outbreak of COVID-19 will happen in your city?				
**5**	How worried are you that you will not be able to go outside of your house if a COVID-19 outbreak happens in your city?				

Section D: Willingness to vaccinate:

1. If there was a free effective vaccine that could prevent COVID-19, would you take it?

a. Yes

b. No

c. Not sure

2. If an effective vaccine is available to prevent COVID-19 with a cost of 10 thousand Yemeni riyals (approximately 15$ at the time of study period), would you take it?

a. Yes

b. No

C. Not sure

If your answer to the previous question (Q2) was “No” or “Not sure,” what factors could prevent you from taking a vaccine to prevent COVID-19, if available?

(Please select all that apply):

-I am not at risk for covid-19, so I do not need it

-The cost of the vaccine (10 thousand Yemeni riyals)

-Concerns about vaccine safety and unexpected side effects

-Lack of confidence in the effectiveness of the vaccine

-I will wait for more studies and results on the vaccine
